# The Forgotten Complication of Nasogastric Tube Insertion: Esophageal Perforation and Associated Hydropneumothorax and Hydropneumoperitoneum

**DOI:** 10.7759/cureus.38699

**Published:** 2023-05-08

**Authors:** Harish Gidda, Mohamed Mansour, Inderpal Singh, Bola Nashed, William Ventimiglia

**Affiliations:** 1 Internal Medicine, Ascension St. John Hospital, Detroit, USA; 2 Pulmonary and Critical Care Medicine, Ascension St. John Hospital, Detroit, USA

**Keywords:** esophageal candidiasis, nutrition support, pulmonary critical care, esophageal rupture, naso gastric tube ngt, gastro-intestinal perforation

## Abstract

Nutritional support is essential for critically ill patients to reduce mortality and length of stay. Frequently nasogastric (NG) tubes are used to provide enteral nutrition. A very rare risk of NG tube placement is esophageal perforation, most commonly in the thoracic portion of the esophagus. Here we describe a case of a 41-year-old male with multiple risk factors for esophageal integrity disruption who initially presented for diabetic ketoacidosis (DKA) requiring intubation. Following intubation, an NG tube was placed for nutritional support. The following day the patient developed hydropneumothorax and hydropneumoperitoneum. He was taken emergently for surgical correction of suspected perforation. It was found that the patient had esophageal perforation from the distal esophagus to the proximal portion of the lesser curvature of the stomach. The NG tube transversed the proximal portion of the tear and re-entered at a distal site. The distal portions of the esophagus showed necrotic superficial layers with viable muscularis layers. The patient gradually improved after surgical intervention and was discharged to a long-term acute care facility. It is essential as medical providers to be familiar with complications of NG tube placement and risk factors that could increase the risk of esophageal perforation.

## Introduction

Nutrition plays an important role in reducing mortality and ICU length of stay [1-2]. Often nasogastric (NG) or nasojejunal (NJ) tubes are placed to provide early nutrition in ventilated patients. The risk of esophageal perforation with NG tube placement is rare, but a known complication, with only a few cases reported [3-4]. A population study conducted in Iceland reported that the incidence of NG tube esophageal perforation was 3.1/1,000,000 cases [5]. The majority of esophageal perforations are iatrogenic [6]. Acute esophageal necrosis (AEN), also called black esophagus, can affect the integrity of the esophageal mucosa, increasing the risk of esophageal rupture with NG tube placement. AEN can occur for various reasons, including diabetic ketoacidosis (DKA), alcohol use, substance use, malnutrition, and critical illness [7-9]. Esophageal candidiasis, another potential infection affecting esophageal mucosal integrity, leading to esophageal perforation, is rare [10-11]. This case report describes a 41-year-old male with multiple risk factors for esophageal compromise, including DKA, esophageal candidiasis, malnutrition, and alcohol and IV drug use. The patient underwent NG tube placement for early nutritional support, complicated by findings of hydropneumothorax and free intraperitoneal air with ascites suggestive of distal esophageal perforation. The patient was taken for emergent surgical intervention where the mucosal layer of the distal esophagus appeared necrotic, with the NG tube transversing the esophagus and re-entering through the distal portion of the perforation. Medical providers must recognize rare but known complications to simple procedures such as NG tube placement. Furthermore, it is crucial to recognize cases where the esophageal structure may be compromised. Earlier recognition of esophageal perforation can lead to an expedited multidisciplinary approach to treating the patient with more successful outcomes.

## Case presentation

**Figure 1:**
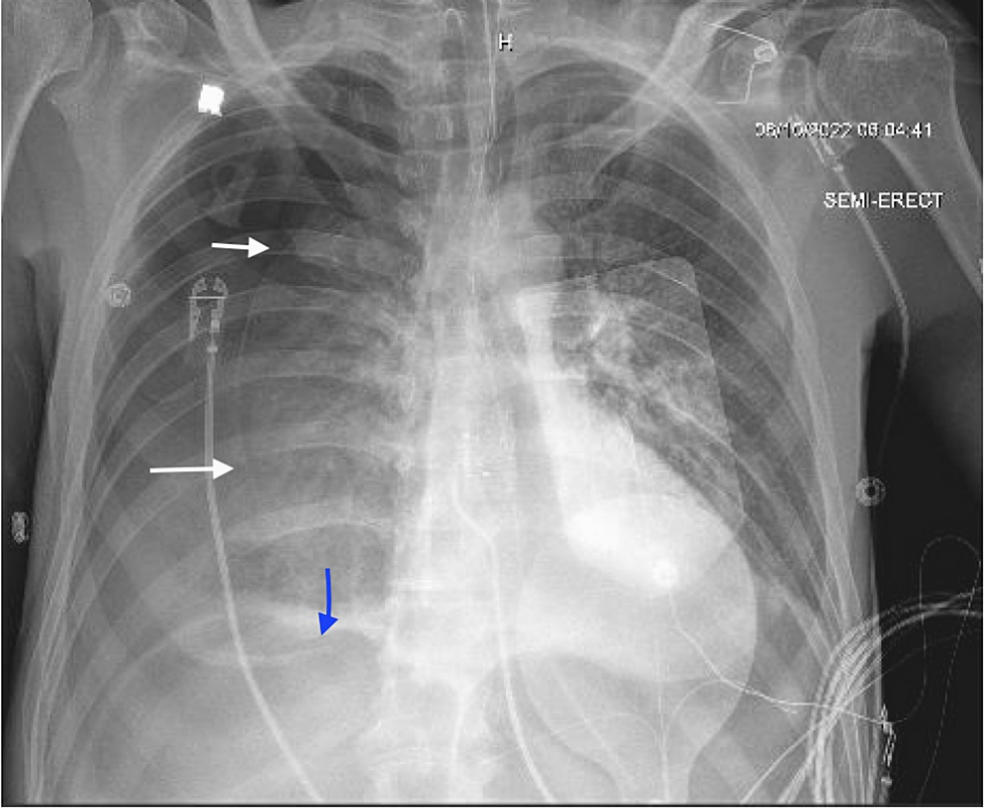
Chest X-ray showing pneumothorax (white arrows) and lucency under the diaphragm, suggestive of free intraperitoneal air (blue arrow).

The case follows a 41-year-old male with a past medical history of IV drug use, alcohol use disorder, history of endocarditis, and type II diabetes mellitus who initially presented with nausea, vomiting, and sepsis without septic shock. He was found to be in DKA with a left shoulder abscess contributing to his sepsis. He was started on vancomycin for his shoulder abscess. The patient was also found to have oropharyngeal candidiasis with suspicion of extension into the esophagus and subsequently started on fluconazole. The patient's worsening acidosis and increasing anion gap persisted due to a delay in initiating insulin, as the potassium level was not determined. He required admission to the ICU. The patient started becoming progressively altered and required intubation and blood pressure support with low-dose Levophed. His antibiotic coverage was broadened to include cefepime. An NG tube was placed for enteral feedings to provide nutrition, confirmed by auscultation. Overnight, the patient required increasing vasopressor support, and two additional vasopressor agents were added with the addition of stress dose steroids. Physical exam showed absent right peripheral breath sounds. Consequently, a chest X-ray showed right hydropneumothorax and free intraperitoneal air (Figure 1).

**Figure 2:**
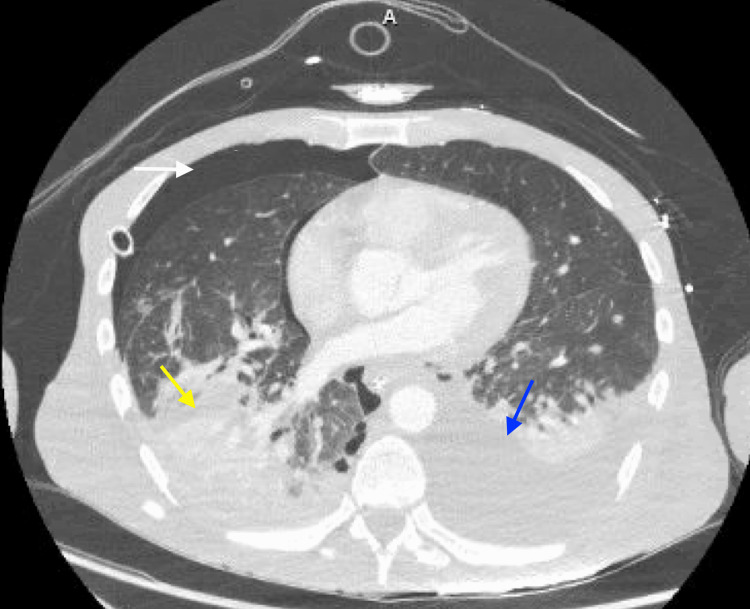
CT of the chest with IV contrast showing pneumothorax (white arrow), right posterior pneumonia (yellow arrow), and left pleural effusion (blue arrow).

**Figure 3:**
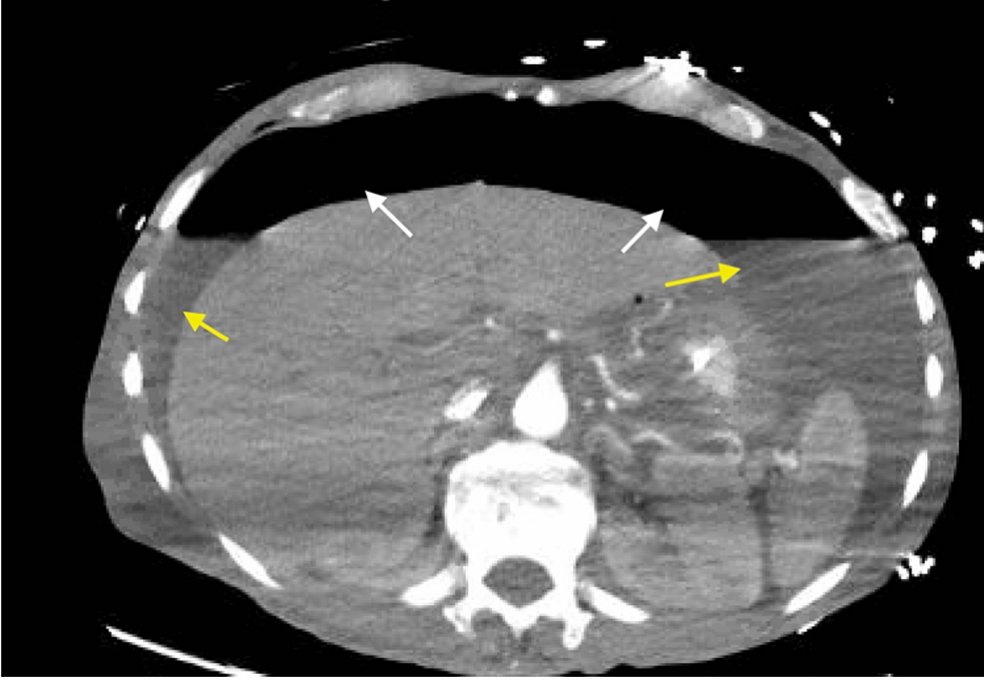
CT of the abdomen with IV contrast showing free intraperitoneal air (white arrows) and ascites (yellow arrows).

A chest tube was placed, draining air and 1L of dark/bilious fluid. There were concerns for esophageal perforation, and a CT chest/abdomen/pelvis with IV, and oral contrast was ordered stat. The CT showed improvement in the right hydropneumothorax with the right chest tube in place. Additionally, the CT showed a large left pleural effusion (Figure 2), large amounts of hydropneumoperitoneum, with small foci of gas near the gastroesophageal junction, and no extravasation of contrast (Figure 3).

The patient was taken emergently for surgery. It was found that the patient had an esophageal tear just above the gastroesophageal junction extending into the proximal lesser curvature of the stomach. The NG tube was visualized protruding through the esophageal tear and back through a distal portion of the tear. Endoscopy in the operating room showed necrotic superficial layers of the distal esophagus with viable muscularis layers. The necrotic material was debrided, and tears were repaired with a suture. Nissen-like fundoplication was then done, wrapped posterior to the stomach and over the repair of the stomach attached to the diaphragm and back to the stomach. A jejunostomy feeding tube was inserted at the time of surgery. No biopsy was done to confirm AEN. Following surgery, the patient had decreased vasopressor requirement. Overall, the patient continually improved. Attempts at spontaneous breathing trials caused the patient to become agitated, tachypneic, and tachycardic. Ultimately, the patient underwent a tracheostomy, and chest tubes were removed. He was then discharged to a long-term acute care facility.

## Discussion

This case report highlights a rare complication of NG tube placement. Often NG and NJ tubes are essential means of nutrition in critically ill patients. The incidence of NG tube esophageal perforation is 3.1/1,000,000 cases [5,11]. The majority of esophageal perforations are iatrogenic [6,11]. It is important to consider this complication with any instrumentation, although it is very rare [5-6]. Esophageal perforations carry high morbidity and mortality. When treated within the first 24 hours, mortality is 10-25% and 40-60% in 48 hours [12]. Diagnostic options include an X-ray assessing for free air or pneumomediastinum, a CT scan of the chest and abdomen, or contrast esophagography. If esophageal perforation is suspected, the patient should be made NPO, and started on proton pump inhibitors, broad-spectrum antibiotics, and antifungals [11]. Often thoracic or abdominal perforations favor surgical repair as part of the initial management [12], as in our case. Other therapeutic options include endoscopic stent placement and surgical drainage [11-12]. Patients will need either a jejunostomy or gastrostomy tube as their esophagus will need time to heal [11]. It is especially important to be observant of patients who have risk factors for esophageal integrity disruption. In this case, the patient had multiple risk factors for esophageal compromise, including alcohol use, substance use, diabetes mellitus, malnutrition, and esophageal candidiasis. Depending on where the esophageal perforation occurs, it can cause hydropneumothorax and pneumomediastinum with expulsions of gastric contents into the lung space or hydropneumoperitoneum if the perforation is below the diaphragm. A delay in recognizing esophageal perforation can result in higher mortality [10,12]. The successful management of this patient required a multidisciplinary approach with long-term care in a long-term acute care facility.

## Conclusions

Nutritional support for critically ill patients must begin as soon as possible to improve outcomes. Frequently NG tubes are used to provide enteral nutritional support. It is important to be aware of factors that may affect esophageal integrity, which may increase the risk of esophageal perforation with NG tube placement. Physicians must be vigilant for these risk factors before placing NG tubes to reduce further the risk of esophageal perforation, which is associated with high mortality and morbidity.
